# Morpho-Agronomic Evaluation of *Lagenaria siceraria* Landraces and Their F_1_ Populations

**DOI:** 10.3390/plants11121558

**Published:** 2022-06-13

**Authors:** Lungani Siyabonga Nkosi, Nontuthuko Rosemary Ntuli, Sydney Mavengahama

**Affiliations:** 1 Food Security and Safety Focus Area, Faculty of Natural and Agricultural Sciences, North-West University, Mmabatho 2745, South Africa; lunganishoes@gmail.com; 2 Department of Botany, University of Zululand, KwaDlangezwa 3886, South Africa; ntulir@unizulu.ac.za

**Keywords:** genotypes, F_1_, qualitative, quantitative traits, genetic variability, fruit, leaf, yield, shape

## Abstract

*Lagenaria siceraria* is one of the most important cucurbitaceous vegetables due to its prolific bearing habit, its edibility as a cooked vegetable, and its low cost of cultivation. The objective of this study was to evaluate variation in the morpho-agronomic traits among selected landraces and their F_1_ populations. The landraces were crossed based on the North Carolina II genetic design to develop F_1_ populations. The twelve F_1_ populations along with seven parental landraces were grown in a randomized complete block design with three replications. Significant differences (*p* < 0.05) were observed among quantitative traits suggesting considerable genetic variability. The genotypes displayed significant variation in most qualitative traits of fruits and seeds. The first five principal components of quantitative traits among the evaluated 19 genotypes contributed 74.84% of the variability. The biplot and dendrogram clustered the genotypes into five clusters according to their vegetative, fruit, and seed traits. The highest value for the broad-sense heritability estimate was recorded for days to edible harvest maturity trait. The F_1_ progenies were more variable than the landraces and can therefore be used for further *Lagenaria siceraria* genetic improvement.

## 1. Introduction

*Lagenaria siceraria* is an important cucurbitaceous vegetable due to its prolific bearing habit, its edibility as a cooked vegetable, and its low cost of cultivation [1]. The primary gene centre of *L. siceraria* is in tropical Africa [2]. Its white flowers and characteristic seed, fruit, and leaf shapes differentiate this crop from other pumpkin varieties [2]. The flower size and monoecious condition make hybridization easy and appropriate [1]. The quantity of cross-pollination ranges between 60 and 80% for this crop [1]. Its landraces in South Africa display great diversity in morphological traits, mostly in fruit shape and size [3]. The valuable tools for the preliminary assessment of genetic variability are morpho-agronomic descriptors because they allow fast insight into the range of diversity that exists [4]. 

Knowledge of genetic diversity in a germplasm collection is important for parental selection in hybridization [5]. Significant genetic diversity occurs in this crop, which can be utilized for the exploitation of hybrid vigor (heterosis) [1]. Heterosis refers to the super performance of a hybrid exhibiting increased biomass, yield, size, growth rate, or fertility relative to its parents [6] and can be attained by crossing parents with complementary traits. It provides a way to overcome the yield barriers [7].

Quantitative variables are useful descriptors, particularly when used to evaluate the agronomic potential of a new cultivar or germplasm accession [4]. The cultivation of *L. siceraria* is largely dependent on indigenous unimproved landraces, which are not scientifically selected and bred, due to the absence of improved cultivars. Since hybrid varieties of *L. siceraria* in Asia have recorded yields of more than 40 t/ha under optimum conditions, while local landraces are reported to produce less than 25 t/ha [8] we hypothesized that this improvement in yield is due to hybridization could also be achieved with the landraces available in South Africa. We, therefore, considered it necessary to select and cross the local landraces that showed desirable traits and evaluate the morpho-agronomic characteristics of their offspring for the future development of new cultivars. The current study was undertaken to evaluate the performance of *Lagenaria siceraria* genotypes (seven parental landraces and their offspring) using morpho-agronomic traits.

## 2. Results

### 2.1. Qualitative Traits Evaluation 

**Figure 1:**
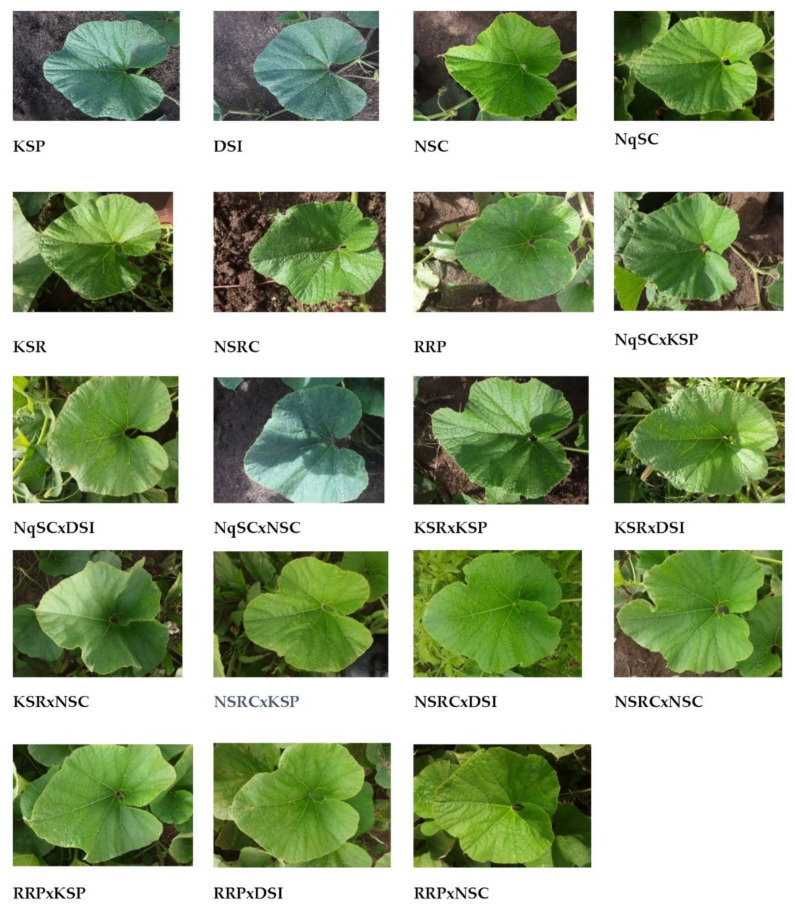
Diversity in leaf color and leaf margin among *Lagenaria siceraria* genotypes.

Spreading was the dominant growth habit exhibited by 95% of the genotypes (18 genotypes), while one genotype (5%) had a bushy growth habit (Table 1). There were differences in leaf margins amongst studied genotypes, with the following types per genotype: crenate (15.8%), spiny (15.8%), sinuate (5.3%), ciliate (10.5%), ciliate-crenate (5.3%), spiny-crenate (10.5%), spiny-sinuate (10.5%), spiny-ciliate (5.3%), spiny-undulate (10.5%), undulate (5.3%), and denticulate (5.3%) margins (Table 1 and Figure 1). The dominant stem shape was angular, displayed by 89% (17 genotypes), while two genotypes (11%) had a round stem shape. Green was the dominant color of leaves, exhibited by 84% (16 genotypes), while three genotypes (16%) exhibited dark green leaf color (Table 1 and Figure 1).

**Figure 2:**
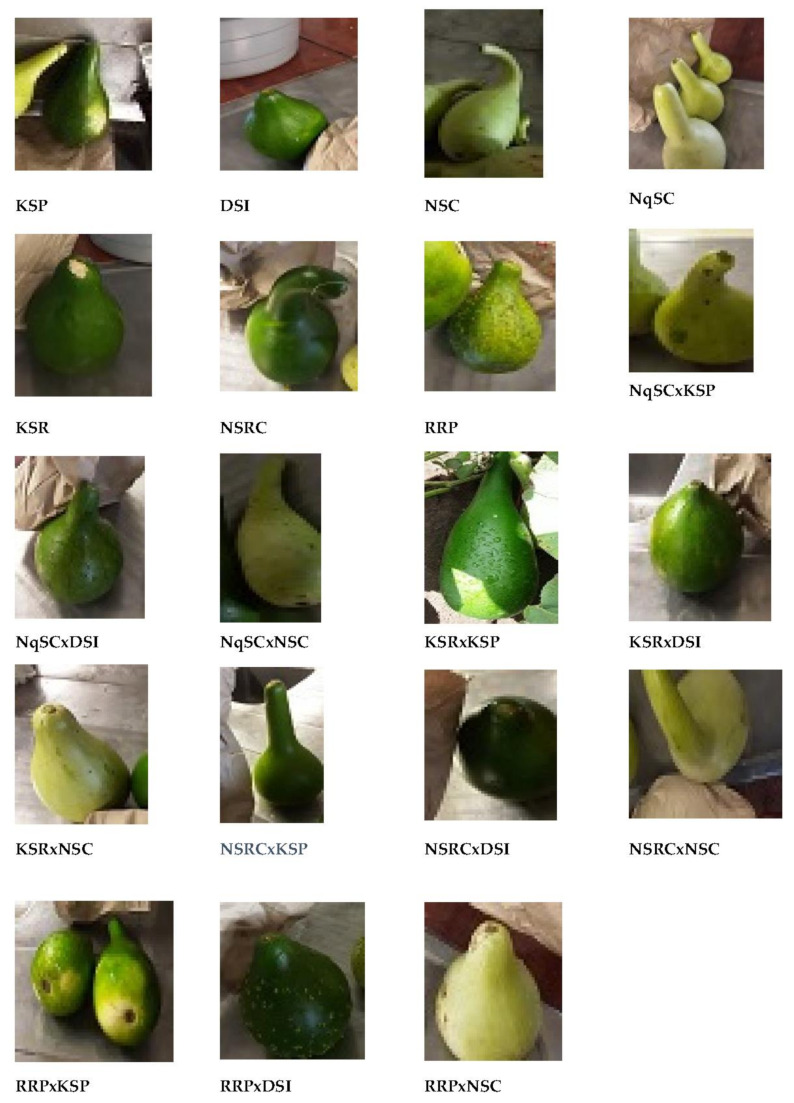
Diversity in fruit color, fruit shape, and fruit texture among *Lagenaria siceraria* genotypes.

The genotypes displayed significant variation in most fruit and seed traits. Some of the parents and offspring produced a mixture of plants that produced either smooth or rough textures or curvilinear and pear shapes resulting in either two textures and shapes being reported for the same genotype (Table 2). The dominant fruit color was dark green exhibited by 47.4% (9) of genotypes, while 36.8% (7) displayed a green color and three (15.8%) had a light green fruit color (Table 2 and Figure 2). Smooth was the dominant fruit texture, exhibited by 57.9% (11) of genotypes. Five (26.3%) of the genotypes displayed both rough and smooth textures and three genotypes (15.8%) had a rough fruit texture (Table 2 and Figure 2). The following differences in fruit shape were recorded amongst studied genotypes, as represented by the number and percentage: curvilinear (4, 21.1%); isodiametric (4, 21.1%); pear (3, 15.8%); pear and isodiametric (2, 10.5%); isodiametric and curvilinear (2, 10.5%); pear and semi-curvilinear (2, 10.5%); semi-curvilinear (1, 5.3%), and; curvilinear and pear (1, 5.3%) shapes (Table 2 and Figure 2).

**Figure 3:**
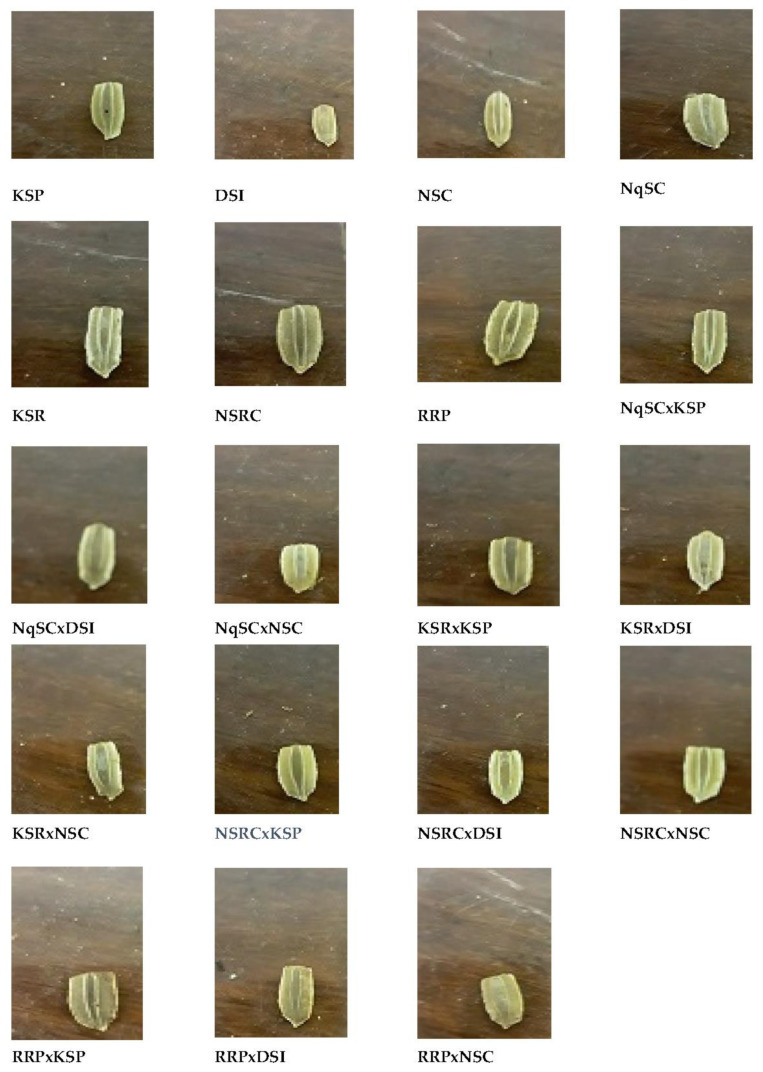
Diversity in seed color and seed shape among *Lagenaria siceraria* genotypes.

The dominant seed color amongst genotypes was brown exhibited by 15 genotypes (78.9%), while three genotypes (15.8%) displayed light brown color and one genotype (5.3%) had dark brown seed color (Table 2 and Figure 3). The recorded differences in the seed shape among genotypes were in the following descending order (number and percentage): ovate (10, 52.6%); oblong (8, 42.1%), and; obovate (1, 5.3%) shapes (Table 2 and Figure 3).

All genotypes exhibited vigorous plants with a rapid ground cover habit, and the tendrils were produced by all genotypes. White flower color, large flower size, green stem color, and white fruit pulp color were observed in all genotypes. Seeds of all genotypes had lines and a rough texture. All genotypes showed a heart-shaped leaf. The tendril type was coiled and branched for all genotypes. There were five petals observed in all genotypes and staminate flowers were exhibited in the main vine, whereas the pistillate flowers were borne in the lateral branches.

### 2.2. Quantitative Traits Evaluation

#### 2.2.1. Seedling Traits

The results displayed significant differences (*p* < 0.05) in some traits. Seedlings of RRP and NSRC genotypes emerged earlier (9 days), while genotype NqSC took longer to emerge (19 days), with a mean of 12 days (Table 3). The KSRxKSP hybrid had a significantly high (81.95%) emergence percentage, whereas genotype NqSC had the lowest (32.92%) with a mean of 61.94% (Table 3). Significantly taller seedlings (a) were obtained with RRP, NqSCxKSP, NqSCxDSI, and NSRCxDSI genotypes but KSP had the shortest (b) and the other genotypes exhibited significant letters of ab (Table 3). Differences were not significant (*p* > 0.05) among all genotypes in terms of cotyledon leaf area (Table 3).

#### 2.2.2. Vegetative Traits

There were insignificant differences (*p* > 0.05) in the number of branches, the number of leaves, and leaf chlorophyll content among all genotypes (Table 3). The tallest vines (143.92 cm) were obtained with RRP, but KSP had the shortest (23.70 cm), with a mean vine length of 101.25 cm (Table 3). RRP exhibited a significantly large leaf area (310.6 cm^2^), while KSP had the smallest (122.9 cm^2^) with a mean of 188.1 cm^2^ (Table 3). Differences were not significant among all genotypes in terms of shoot growth percentage (Table 4). A significantly higher leaf growth percentage (a) was obtained with KSP, DSI, NSC, NqSC, KSR, NSRC, NqSCxNSC, NqSCxDSI, KSRxKSP, KSRxDSI, KSRxNSC, NSRCxKSP, NSRCxDSI, NSRCxNSC, and RRPxNSC genotypes, but NqSCxKSP had the lowest (b) and the other genotypes (RRP, RRPxKSP and RRPxDSI) exhibited significant letters of ab (Table 4).

#### 2.2.3. Reproductive Traits

The NSRCxKSP, NSRCxDSI, NSRCxNSC, KSRxKSP, RRPxKSP, and RRPxDSI crosses exhibited significantly (*p* < 0.05) fewer days to flowering, while genotype KSP took longer to flower (Table 3).

#### 2.2.4. Fruit Yield and Agronomic Traits at Harvest

The crosses NSRCxKSP and RRPxKSP exhibited significantly fewer days to edible harvest maturity (74 days), while genotype DSI and KSRxKSP took longer to reach edible harvest maturity (80 days) with a mean of 77.89 days. The NSRCxKSP cross had significantly fewer days to drying harvest maturity, while genotypes DSI, KSP, NSC, NqSC, and KSR took longer to reach drying harvest maturity. Differences were not significant (*p* > 0.05) among all genotypes in terms of fruit yield per plot, fruit mass, fruit width, fruit length, total fruit mass per plot, fruit rind thickness, yield per plant, and the number of fruits per plant. Fruit neck length ranged significantly from 2.07 cm (KSRxDSI) to 11.60 cm (NSC) with an average of 7.75 cm (Table 4).

#### 2.2.5. Seed Traits

Differences were not significant (*p* > 0.05) among all genotypes in terms of one hundred seed mass, total seed mass, seed length and the number of seeds per fruit. Seed width varied significantly from 7.50 mm for DSI to 10.67 mm for RRP with a mean of 9.56 mm (Table 4).

### 2.3. Correlation among Traits

Emergence percentage correlated positively with the number of seeds per fruit but was negatively correlated with the number of fruits per plant. Positive correlations were observed between number of branches with a hundred seed mass, total seed mass, fruit width, seedling height, cotyledons leaf area, leaf area, vine length, the number of leaves, and seed width. The total seed mass was positively associated with seedling height, hundred seed mass, vine length, the number of leaves, the number of seeds per fruit, and the cotyledon leaf area. Leaf growth percentage was negatively correlated with total seed mass. Fruit yield correlated positively with the seedling height. Fruit rind thickness correlated positively with seedling height, hundred seed mass, vine length, fruit width, and leaf area. Fruit rind thickness correlated negatively with the number of fruits per plant, fruit neck length, and leaf growth percentage (Table 5).

Positive correlations were observed between the number of leaves with hundred seed mass (r = 0.68), seed width (r = 0.70), and fruit width (r = 0.57). The number of leaves correlated negatively with leaf growth percentage (r = −0.68). Hundred seed mass correlated positively with number of seeds per fruit (r = 0.58), fruit length (r = 0.51), fruit width (r = 0.69), and seed width (r = 0.49). A negative correlation was observed between hundred seed mass and leaf growth percentage (r = −0.67). Fruit length correlated positively with fruit neck length (r = 0.74) and, again, fruit length significantly correlated negatively with leaf growth percentage (r = −0.49). Fruit width correlated negatively with leaf growth percentage (r = −0.65).

### 2.4. Principal Component Analysis

There was diversity among genotypes identified by principal component analysis (Table 6). The first five components contributed 74.84% of the variability. Emergence percentage, seedling height, cotyledon leaf area, leaf area, vine length, number of leaves, number of branches, number of seeds per fruit, fruit mass, fruit rind thickness, hundred seed mass, total seed mass, fruit length, fruit width, seed width, fruit yield, yield per plant, leaf chlorophyll content, total fruit mass per plot, fruit neck length, and seed length were correlated positively with the first principal component (PC1), which accounted for 34.46% of the total variation. Days to emergence, days to flowering, days to edible harvest maturity, days to drying harvest maturity, number of fruits per plant, leaf growth percentage, and shoot growth percentage correlated negatively with PC1.

The days to emergence, days to flowering, days to edible harvest maturity, days to drying harvest maturity, shoot growth percentage, cotyledon leaf area, leaf area, vine length, number of leaves, number of branches, fruit mass, fruit rind thickness, hundred seed mass, fruit width, seed length, and seed width associated positively with PC2, which accounted for 14.48% of the total variability. Emergence percentage, leaf chlorophyll content, total fruit mass per plot, number of seeds per fruit, number of fruits per plant, total seed mass, fruit length, fruit neck length, leaf growth percentage, fruit yield, and yield per plant correlated negatively with PC2. There was no strong correlation identified by PC1 and PC2 in all the evaluated morphological traits.

### 2.5. Cluster and Biplot Analysis

**Figure 4:**
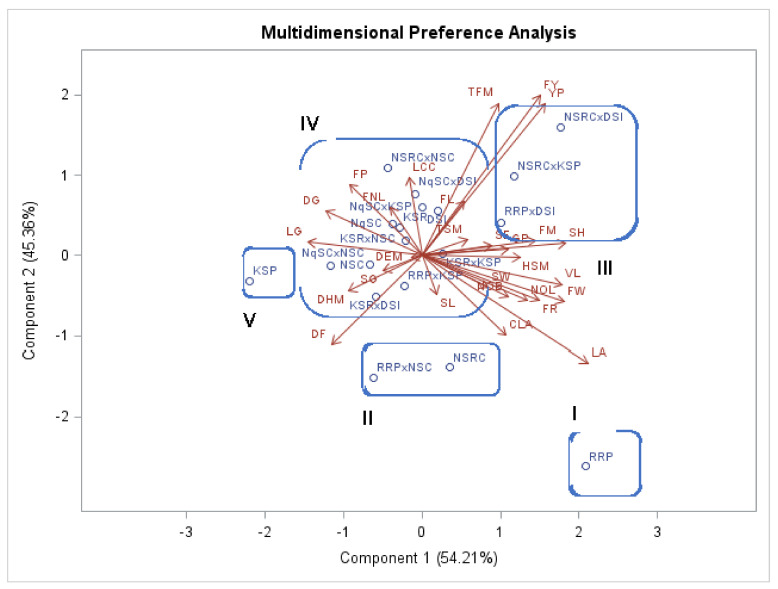
Biplot of *Lagenaria siceraria* genotypes and agronomic traits. Genotypes and agronomic traits (variables) are described in Table 2 and Table 3 and Table 4, respectively.

**Figure 5:**
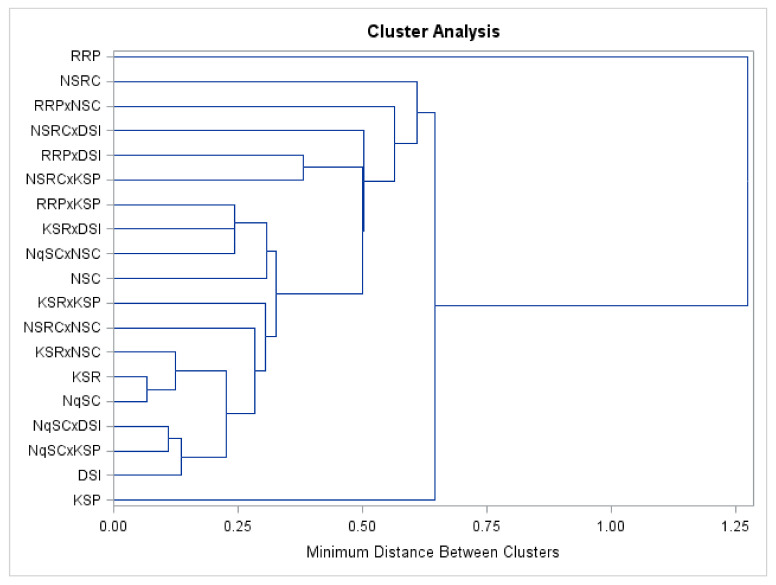
Hierarchical cluster showing similarities amongst *Lagenaria siceraria* genotypes using the complete linkage method. The description of genotypes appears in Table 2.

Evaluated genotypes clustered according to their vegetative, fruit, and seed traits as shown in the biplot (Figure 4) and dendrogram (Figure 5). The first Cluster (I) in the biplot and Group I of the dendrogram consisted of the RRP genotype with tall seedlings, large cotyledon size, least shoot growth percentage, a high number of branches, a high number of leaves, large leaf area, long vine length, large rind thickness, and large seed width. RRP fruits were green in color, rough in texture, isodiametric shaped, and had white pulp, whereas the seeds were brown, rough, ovate, large, and had lines. The second Cluster (II) in the biplot and Group II of the dendrogram consisted of the RRPxNSC and NSRC genotypes with similar vine length, leaf chlorophyll content, total fruit mass per plot, and leaf growth percentage. Fruits of these genotypes were smooth with white fruit pulp, while the seeds were rough with lines. The third Cluster (III) in the biplot and Group III of the dendrogram consisted of the RRPxDSI, NSRCxDSI, and NSRCxKSP genotypes with similar leaf area and seed width. Fruits of these genotypes exhibited white pulp and the seeds were rough, oblong, and had lines.

The fourth Cluster (IV) in the biplot and Group IV of the dendrogram consisted of the RRPxKSP, NSRCxNSC, KSRxDSI, NqSCxNSC, NSC, KSRxKSP, KSRxNSC, NqSC, NqSCxDSI, NqSCxKSP, DSI, and KSR genotypes with similar fruit width, a low number of leaves, and small leaf area and seed length. The fruits of these genotypes were smooth with white pulp, whereas the seeds were brown, rough, and with lines. The fifth Cluster (V) in the biplot and Group V of the dendrogram consisted of the KSP genotype with short seedlings, small cotyledon size, high leaf growth percentage, low number of branches, low number of leaves, small leaf area, short vine length, small rind thickness, and low fruit yield. KSP fruits were dark green, smooth, pear-shaped with white pulp and the seeds were brown, rough, oblong, and small in size with lines.

### 2.6. Genetic Parameters

In the current study, both phenotypic variance and phenotypic coefficient of variation were generally higher than genotypic variance and genotypic coefficient of variation. The genetic parameters are shown in Table 7.

## 3. Discussion

### 3.1. Choice of Parents Crossed in the Study

The landraces were named according to their origin and fruit morphology [9]. Four landraces that were used as male parents were the KSR from Khangelani, NSRC and NqSC from Nquthu, as well as the RRP from Rorke’s Drift. Three that were used as female parents were DSI from Dundee, KSP from Khangelani, and NSC from Nquthu. The female parents were selected based on their bigger average number of pistillate flowers than the male parents, whereas the male parents were selected based on their high number of staminate flowers than the female parents [9]. Fruit shape and fruit texture were also considered in selecting the female parents and the male parents for the possible mixing of traits in the generated F_1_ offspring.

### 3.2. Qualitative Traits Evaluation

The 19 genotypes (seven parental landraces and 12 F_1_ progenies) evaluated in the present study showed a wide range of diversity in qualitative traits including growth habit, stem shape, leaf color, leaf margin, fruit color, fruit texture, fruit shape, seed color, seed shape, and seed size (Table 1 and Table 2). Related findings were reported on *L. siceraria* landraces characterization, where the greatest variation was in fruit shape, fruit texture, fruit color, seed size, seed shape, and seed color [10]. The present results revealed that different landraces display different morphology. However, plant vigor, tendrils, stem color, leaf shape, flower color, flower size, tendrils branching, tendril type, fruit pulp color, seed texture, and seed lines were constant for all evaluated genotypes. Corresponding findings were reported by Kalyanrao et al. [10] who observed that *L. siceraria* genotypes were similar in stem color, tendrils, tendril branching, leaf shape, and flower color but differ in fruit and most seed traits.

Observed diversity in fruit and seed qualitative traits among the genotypes in the present study could be due to the different sites of origin of the seven landraces crossed and the mixing of genes in the controlled cross-pollination performed for generating 12 first filial generations (F_1_ progenies). It was observed that the F_1_ offspring inherited either the male or female fruit morphological traits (fruit color, fruit texture, and fruit shape) in the current findings. For example, the cross between the DSI (maternal parent) with dark green smooth isodiametric fruits and the RRP (paternal parent) with green rough isodiametric fruits generated green rough isodiametric fruits of the RRPxDSI (F_1_ progeny).

### 3.3. Quantitative Traits Evaluation

Seven landraces and twelve F_1_ progenies evaluated in the present study showed a wide range of diversity in quantitative traits including days to emergence, emergence percentage, seedling height, leaf area, main vine length, days to flowering, days to edible harvest maturity, days to drying harvest maturity, fruit neck length, seed width, and leaf growth percentage. There was no significant difference for days to 50% emergence in a study by Mashilo et al. [11] in *Lagenaria siceraria* landraces collected from Limpopo, whereas in the present study there was a significant difference in days to emergence among the 19 evaluated genotypes. The variation observed in the days to emergence was mainly due to differences in sites of origin for the landraces. In the current study, two landraces (RRP and NSRC) reached 50% emergence before all F_1_ offspring emerged and one parental line (NqSC) emerged later than all F_1_ progenies. Similar findings were reported on the significance of emergence percentage among *L. siceraria* genotypes that ranged from 65.8% to 85.3% [12].

The highest emergence percentage was obtained from F_1_ progeny (KSRxKSP), while the lowest was observed from the parental line (NqSC). This means F_1_ progenies displayed heterosis, where the progeny of a cross performs better than either parent. Accessions of *L. siceraria* collected from Turkey displayed the same range in seedling height (22–56 mm), but their mean (39 mm) was lower than the one obtained in the present study [3]. This observation confirms that different growing environments affect the performance of *Lagenaria siceraria* populations. Buthelezi et al. [9] obtained lower cotyledon leaf areas which ranged from 880–1726 mm^2^ and a mean of 1289 mm^2^ amongst the landraces of *L. siceraria*. The diversity in seedling traits obtained in the current study may be due to the genetics of different *L. siceraria* genotypes [13].

A shorter main vine length was obtained in the present study than reported amongst *Lagenaria siceraria* lines (4.8 to 6.8 m) by Uddin et al. [13]. In the present study, we observed a larger leaf area (2581 to 7000 mm^2^) than the results reported on *L. siceraria* landraces by Mashilo et al. [11]. The findings obtained in the current study agree with the observations on days to flowering in *L. siceraria* that ranged from 44.0 to 65.8 days from a study by Iqbal et al. [12]. The current findings on days to drying harvest maturity correspond with published findings that *L. siceraria* reaches maturity from 60–120 days after planting [14]. Mashilo et al. [11] reported a longer maximum fruit neck length (24.1 cm) and shorter minimum fruit neck length (0.53 cm) than the present study amongst *L. siceraria* landraces. Thus, different genotypes differ in the fruit neck length which affects their usage when the fruits are dry. For example, isodiametric-shaped fruits have a short neck, therefore the matured dried fruits are used for storing water and milk. The dried curvilinear shaped fruits with long necks are used as cups for traditional beer in rural communities.

Results on seed width from the present study agreed with a reported study on African *L. siceraria* accessions, where the seed width ranged from 5.30 to 12.34 mm [2]. A greater *L. siceraria* leaf growth percentage with a mean of 5281% was recorded in the previous study by Buthelezi et al. [9]. However, cotyledon leaf area, number of leaves, number of branches, leaf chlorophyll content, total fruit mass per plot, number of seeds per fruit, fruit mass, fruit rind thickness, hundred seed mass, total seed mass, fruit length, fruit width, seed length, shoot growth percentage, fruit yield, and yield per plant were not significantly different (*p* > 0.05) for the evaluated genotypes in the current study. These results of the present study disagree with reported results, where significant differences (*p* < 0.05) were obtained in most quantitative traits of *Lagenaria siceraria* [11].

The positive correlation between emergence percentage with fruit yield, number of seeds per fruit, and vine length suggests that the selection of genotypes with high emergence percentage results in genotypes with higher fruit yield, many seeds per fruit, and long vines. A positive correlation was observed in the current study between the number of seeds per fruit with the vine length, the number of branches, and total fruit mass, which is in accordance with the reported findings [15]. Similar correlations were reported between the number of branches and the vine length h (r = 0.614) and the number of leaves (r = 0.678) [9].

A comparable positive correlation was obtained by Mashilo et al. [11] between hundred seed mass and the vine length (r = 0.45) and the number of branches (r = 0.42). Seed length was also reported to be positively correlated with fruit length and fruit rind thickness which also correlated positively with fruit circumference in the comparable study [2]. The present results are like those of Buthelezi et al. [9] who also reported that seed mass positively correlated with hundred seed mass (r = 0.966) and fruit width (r = 0.732); fruit yield was also reported to correlate positively with fruit width (r = 0.702) and seed width was also reported to be positively correlated to tendril traits. Total fruit mass positively correlated with all other variables studied on *L. siceraria* accessions in a study by Mlandenovic et al. [2].

The first principal component (PC1) included several traits that contributed to a higher variation than PC2, as shown by the principal component analysis of the quantitative traits studied. Fruit width and fruit mass were negatively correlated with the first principal component. In the first and second principal components, the traits with high coefficients were considered more important for the explanation of total variability. Seedling height, cotyledon size, vine length, leaf area, number of leaves, number of branches, fruit mass, fruit rind thickness, fruit width, hundred seed mass, and number of seeds per fruit established the greatest variability among the genotypes. Mashilo et al. [11] reported comparable findings, where the principal component analysis indicated that most of the variation in *L. siceraria* is contributed by fruit and seed traits.

Clustering of genotypes according to fruit texture agreed with a previous study conducted on *Lagenaria siceraria* landraces by Buthelezi et al. [9]. It was reported that genotypes with analogous morphology belong to one cluster [8] and that genotypes with identical fruit shape, fruit length, fruit mass, and fruit circumference grouped together [2]. Cluster analysis conducted on quantitative traits grouped the evaluated genotypes into five clusters showing an adequate heritable variation that could permit rational selection.

The genotypic coefficient of variation was generally lower than the phenotypic coefficient of variability signifying a strong impact of the environment concerning the expressiveness of genes in the phenotypic display (Table 7). Supportive results were obtained in a study conducted on the genotypes of *Pisum sativum*, where the genotypic coefficient was generally lower than the phenotypic coefficient of variability [16]. A higher coefficient of variation was obtained with leaf growth percentage in the current study, but pod bearing length exhibited a higher value in the study conducted on field pea by Meena et al. [16]. Higher heritability estimates were recorded for fruit length, vine length, seed width, fruit weight, rind thickness, leaf area, the thickness of seed, number of seeds per plant, and fruit circumference in another study [2]. In most of the traits evaluated in the current study, the genetic advance was lower than the one obtained in a study conducted on *Corchorus* accessions by Dube et al. [17].

## 4. Materials and Methods

### 4.1. Planting Material

The planting materials for this study consisted of seven *Lagenaria siceraria* landraces as the parents and the twelve F_1_ progenies. The seven landraces (Table 8) were collected from various geographic locations in KwaZulu-Natal, South Africa. The selection was based on their early growth, high yield, and the high number of flower traits, following a study conducted by Buthelezi et al. [9]. These landraces were collected from Dundee (28.1650° S, 30.2343° E), Khangelani (29.0106° S, 31.2211° E), Nquthu (28.2195° S, 30.6746° E), and Rorke’s Drift (28.3492° S, 30.5351° E) [9].

### 4.2. Experimental Site

A field experiment for the first season was conducted at North-West University (NWU) crop science field (25°49′34″ S, 25°36′34″ E) from October 2020 to January 2021. The second season field experiment was conducted at Molelwane North-West University research farm, (25°48′ S, 25°38′ E) from January to May 2021. Both sites are located in Mafikeng, South Africa. The area usually receives a mean annual rainfall of 571 mm during the summer seasons [18]. The temperature ranges from an average minimum of 7 °C in winter to an average maximum of 37 °C in January. Soil samples were collected (0–30 cm) before planting and sent to the analytical laboratory of the KwaZulu-Natal Department of Agriculture and Rural Development for analysis, using the rapid procedures described by Hunter [19]. The soils of the site belong to the Hutton series according to the South African Soil Classification, with loamy sand to sandy clay loam texture [18].

### 4.3. Experimental Design and Field Establishment

#### Crossing

The crossing study was carried out in pots under semi-controlled environmental conditions (net house) at North-West University (25°49′34″ S, 25°36′34″ E) from November 2019 to April 2020. The soil to fill the ten-liter pots of 30 cm diameter was collected from the crop science research field. The soil was sieved using a 5 mm sieve before filling the pots. Three seeds were planted per pot. Fertilizer NPK 13: 7: 10 (30) + 0.5% Zn + 5% S + 3% Ca at a rate of 3.6 g/plant was mixed with the soil thoroughly in each pot as basal dose before sowing. Limestone Ammonium Nitrate (LAN 28% N) was applied with irrigation water where 3.6 g was dissolved in one litre of water and applied per plant one month after planting. At flowering, the same rate of LAN fertigation was applied to avoid cutting off roots. Pots were hand-weeded and the plants were sprayed with Malasol (active ingredient: Mercaptothion 500 g/L) to control pests and irrigation water was supplied where necessary

At planting, the temperature was 28 °C in the net house, whereas outside it was 31 °C with 0% precipitation. Laboratory thermometers were used to measure the temperature in the net house. The average minimum day temperature was 16 °C and the average maximum day temperature was 31 °C in the net house during the growing season. The pots were labelled for identification and after planting they were irrigated adequately.

The selected landraces were crossed based on the North Carolina II genetic design to develop F1 progenies. Blocking was performed in this study so as to allow all mating involving a single group of males to a single group of females to be kept intact as a unit (Table 9) [20]. The pistillate flowers of the female parent and staminate flowers of the male parent were bagged separately a day before opening and hand pollination was done the next morning immediately followed by bagging for two to three days. The cross-pollination was performed early in the morning (before 06:00 a.m.) using ear buds to transfer pollen grains from the staminate flower to the pistillate flower. An earbud was used once for each crossing and discarded afterward. The pollinated flowers were tagged for identification.

### 4.4. Field Evaluation

The twelve F_1_ progenies along with seven landraces were grown in a randomized complete block design with three replications. The field was divided into 19 equal-sized gross plots with three replicates per genotype, which made a sum of 57 gross plots in total. Each gross plot was 9 m^2^ (3 × 3) with 1 m inter-row spacing and 1 m intra-row spacing, resulting in 16 plants per plot. There were four rows per gross plot. The spacing between plots was 1.5 m. The seeds were sown directly into the soil at 5 cm depth.

Soil analysis was conducted before sowing. Fertilizer NPK 13: 7: 10 (30) + 0.5% Zn + 5% S + 3% Ca at a rate of 40 g/m^2^ was mixed with the soil thoroughly in each plot in equal amount to the basal dose before sowing. Nitrogen fertilizer Limestone Ammonium Nitrate (LAN 28% N) at the same rate was broadcasted around each plant, one month after planting, and the second one was given at flowering. The field was irrigated to field capacity after fertilizer application and the supplementary irrigation was applied three times a week until maturity. Weed control was performed manually using hand-hoes, and Malasol (active ingredient: Mercaptothion 500 g/L) was used to control aphid infestations.

### 4.5. Data Collection

#### 4.5.1. Seedling Traits

Days to emergence were counted from the date of seeding until 50% of the plants had emerged and the mean was calculated. The emergence percentage was calculated by counting the total number of plants that emerged in each plot and the emergence percentage was calculated with the formula [12].

$$\mathrm{Emergence}\;\mathrm{percentage}\;=\;\frac{\mathrm{Number}\;\mathrm{of}\;\mathrm{seedlings}\;\mathrm{emerged}}{\mathrm{Number}\;\mathrm{of}\;\mathrm{seeds}\;\mathrm{planted}}\;\times \;100$$

The Emergence percentage was calculated from 16 seeds sown per plot in the field. The seedling height (cm) and cotyledon size (mm^2^) were measured at the first true leaf stage using a 300 mm ruler. Height was measured from the ground surface to the apical growth tip of the seedlings. Cotyledon size was measured on one cotyledon leaf in three seedlings per plot using the formula: Cotyledon leaf area (cm^2^) = length (cm) × width (cm) × 0.88 [21].

#### 4.5.2. Vine Traits

The apical point of the desired vines was marked with the artline90^®^ permanent marker just below the first true leaf. At 43 days after planting (DAP), the initial shoot length (from the base of the marker towards the apical tip of the leaf) and initial leaf area (length × width × 0.88) of the first true leaf were measured. At 50 days after planting, the final shoot length was measured from the base of the initial point towards the apical end of the shoot. At 50 DAP, the final leaf area (length × width × 0.88) of the first true leaf was measured. Shoot and leaf growth was calculated using the formula: Growth (%) = ((final − initial) ÷ initial) × 100 [9]. Three net plants were selected randomly from each plot and the length of the main vine (cm) was measured with a measuring tape and a string (which traced the vine length due to its flexibility properties) and the mean was calculated. A measuring tape was used to measure the vine length from the stem base to the leaf apex. On the same vines that were measured for length, the number of branches and number of leaves were also counted. The leaf area (cm^2^) was measured in three net plants per plot using the formula: LA = 0.88 LW [21]. The following qualitative traits were recorded, namely early plant vigor, plant growth habit, stem shape, presence or absence of tendrils, tendril type, tendril branching, stem color, the color of leaves, leaf shape, and leaf margin.

#### 4.5.3. Chlorophyll Content Index

The leaf chlorophyll content was taken from three plants per plot using the chlorophyll meter (model CCM-200 plus) in chlorophyll content index (cci) units. CCM-200 plus output, expressed in chlorophyll content index, is the ratio of radiation transmission from a light-emitting diode centered at 931 nm to the radiation transmitted from the light-emitting diode centered at 653 nm [22]. The data was collected from the third true leaf, where one reading was recorded per leaf.

#### 4.5.4. Flower Traits

The number of days to male and female flowering was counted from the date of emergence to the appearance of 50% of flowers and the mean was calculated. At the flowering stage, the number of staminate and pistillate flowers from three selected plants per plot for each of the three replicate plots (a total of nine plants per genotype) were recorded. Following Buthelezi et al. [9], the flower sex ratio was calculated using the formula: Flower sex ratio (FSR) = (number of staminate flowers ÷ number of pistillate flowers). The following flower traits were recorded: location of flowers on the plant, flower color, flower size, and the number of petals.

#### 4.5.5. Days to Fruit Maturity, Fruit Yield, and Agronomic Traits at Harvest

The number of days was counted from the date of sowing to fruit maturity and the mean was calculated. Fruits were considered mature when they were still green in color, but with a hard rind. At fruit maturity, the plants lost most of their leaves. The number of fruits per plot was recorded at maturity. The number of fruits per plant was recorded from three randomly selected plants from each net plot at every picking. The total number of fruits picked was counted and then averaged to the number of fruits per plant. Fruit mass was measured using the analytical weighing scale in grams (KERN KB 10000-1N) and fruit size was measured using a Vernier calliper to measure the length (cm) and width (cm) of the fruits. The three fruits were selected randomly from harvested fruits of each plot and weighed with an analytical weighing scale and their mean was determined to find the fruit mass.

The total mass of all the harvested fruits per plot from each picking was weighed and the fruit yield per hectare was calculated. Following Oloyede et al. [23], the fruit yield per hectare was calculated using the following formula:

$$\mathrm{Fruit}\;\mathrm{yield}\left(\mathrm{kg}/\mathrm{ha}\right)\;=\;\frac{\mathrm{fruit}\;\mathrm{mass}/\mathrm{plot}\;\left(\mathrm{kg}\right)\times 10,000\;m^{2}}{\mathrm{Area}\;\mathrm{of}\;\mathrm{the}\;\mathrm{plot}\left(m^{2}\right)\times 1\;\mathrm{kg}\;}$$

The cutting of fruits longitudinally was performed, and the rind thickness (mm) was measured using a Vernier calliper. Fruit neck length was measured in cm with a Vernier calliper. The following qualitative traits were recorded: fruit color, fruit shape, fruit texture, and fruit pulp color.

#### 4.5.6. Seed Size and Mass

At harvest, the seeds were removed from the fruit pulp and were air-dried at room temperature (25 °C) for one week. A Vernier calliper was used to measure the seed length (mm) and width (mm). One hundred seed mass and total seed mass from three fruits per plot were measured using the analytical weighing scale in grams (KERN KB 2000-2N) for each of 19 genotypes (12 F_1_ progenies and seven landraces). The following traits were recorded, namely, the number of seeds per fruit, seed size, seed color, seed shape, seed texture, and the presence or absence of seed lines.

### 4.6. Statistical Analysis

The data collected were subjected to analysis of variance (ANOVA) using the general linear model (GLM) of the Statistical Analysis System program (SAS software version 9.4) [24]. The differences between male and female parents and their interactions were measured based on Tukey’s HSD test at a 5% significance level. Correlations and principal component analysis (PCA) were performed to determine multi-character variation. Cluster analysis through a biplot and dendrogram was performed to study the differences and similarities between landraces and F_1_ progenies.

### 4.7. Variance Components Estimation

The genotypic, phenotypic, and environmental variances and the coefficient of variation were calculated according to the formulas described by Burton and Devane [25] as follows:

- Environmental variance (δ^2^e) = MSE
- Genotypic variance (δ^2^g) = $\frac{\left(\mathrm{MSG}-\mathrm{MSE}\right)}{\mathrm{rs}}$
- Phenotypic variance (δ^2^p) = δ^2^g + δ^2^e

where MSG is the mean square due to genotype, MSE is the mean square of error (environmental variance), and (r) is the replications number.

- Phenotypic coefficient of variation (PCV) = $\frac{\sqrt{\delta^{2}p}}{X}\;\times 100$
- Genotypic coefficient of variation (GCV) = $\frac{\sqrt{\delta^{2}g}}{X}\;\times 100$

where: δ^2^p = phenotypic variation, δ^2^g = genotypic variation, X = grand mean of the character to be studied. Estimation of heritability in the broad-sense: Broad-sense heritability (H^2^) expressed as the percentage of the ratio of the genotypic variance (δ^2^g) to the phenotypic variance (δ^2^p), according to Allard [26], was calculated with the following formula:

- H^2^ = $\frac{\delta^{2}g}{\delta^{2}p}$

Genetic advance (GA) was estimated as per the formula given by Allard [26].

$$\mathrm{GA}\;=\;\left(k\right)(\sqrt{\delta^{2}p})(H^{2})$$

where: GA = expected genetic advance, δ^2^p = phenotypic variation, δ^2^g = genotypic variation, and k = the standard selection differential at 5% selection intensity (k = 2.063).

## 5. Conclusions

Wide variation exists in morpho-agronomic traits of the studied *Lagenaria siceraria* landraces and their F_1_ progenies. In the current study, it was observed that the F_1_ offspring inherited either the male or female fruit morphological traits. For example, the cross between the DSI with dark green smooth isodiametric fruits and the RRP with green rough isodiametric fruits generated green rough isodiametric fruits of the RRPxDSI. F_1_ progenies had superior performance than landraces in 15 quantitative traits (53.57%) out of the 28 evaluated traits. Although not statistically different, Cluster III genotypes (NSRCxDSI, NSRCxKSP, and RRPxDSI) outperformed all genotypes with respect to high fruit yield, total fruit mass per plot, individual fruit mass, and large fruit width. Hence, these F_1_ progenies can be used for further *Lagenaria siceraria* genetic improvement.

## Figures and Tables

**Table 1:** Variation in vegetative and reproductive qualitative traits among *Lagenaria siceraria* genotypes.

Genotype	Growth Habit	Stem Shape	Leaf Color	Leaf Margin
KSP	spreading	Angular	Dark Green	Crenate
DSI	Spreading	Angular	Dark Green	Spiny
NSC	Bushy	Angular	Green	Sinuate
NqSC	Spreading	Angular	Green	Spiny
KSR	Spreading	Angular	Green	Ciliate
NSRC	Spreading	Angular	Green	Crenate
RRP	Spreading	Angular	Green	Ciliate-Crenate
NqSCxKSP	Spreading	Angular	Green	Spiny
NqSCxDSI	Spreading	Angular	Green	Spiny-Crenate
NqSCxNSC	Spreading	Angular	Dark Green	Spiny-Sinuate
KSRxKSP	Spreading	Angular	Green	Spiny-ciliate
KSRxDSI	Spreading	Round	Green	Spiny-undulate
KSRxNSC	Spreading	Angular	Green	Spiny-sinuate
NSRCxKSP	Spreading	Round	Green	Ciliate
NSRCxDSI	Spreading	Angular	Green	Undulate
NSRCxNSC	Spreading	Angular	Green	Spiny-crenate
RRPxKSP	Spreading	Angular	Green	Denticulate
RRPxDSI	Spreading	Angular	Green	Spiny-undulate
RRPxNSC	Spreading	Angular	Green	Crenate

**Table 2:** Description of *Lagenaria siceraria* genotypes according to the fruit and seed morphology.

Genotype	Fruit Color	Fruit Texture	Fruit Shape	Seed Color	Seed Shape
KSP	Dark green	Smooth	Pear	Brown	Oblong
DSI	Dark green	Smooth & rough	Isodiametric	Brown	Oblong
NSC	Light green	Smooth	Curvilinear	Brown	Ovate
NqSC	Green	Smooth	Semi-Curvilinear	Brown	Ovate
KSR	Dark green	Rough	Pear	Brown	Oblong
NSRC	Dark green	Smooth	Curvilinear	Light brown	Obovate
RRP	Green	Rough	Isodiametric	Brown	Ovate
NqSCxKSP	Green	Smooth	Curvilinear & Pear	Brown	Ovate
NqSCxDSI	Dark green	Smooth & rough	Isodiametric & curvilinear	Brown	Ovate
NqSCxNSC	Light green	Smooth	Curvilinear	Brown	Ovate
KSRxKSP	Dark green	Smooth	Pear	Brown	Oblong
KSRxDSI	Dark green	Smooth & rough	Isodiametric	Brown	Oblong
KSRxNSC	Green	Smooth	Pear & semi-curvilinear	Brown	Ovate
NSRCxKSP	Dark green	Smooth	Pear & semi-curvilinear	Light brown	Oblong
NSRCxDSI	Dark green	Smooth & rough	Pear & isodiametric	Brown	Oblong
NSRCxNSC	Green	Smooth	Curvilinear	Light brown	Ovate
RRPxKSP	Green	Smooth & rough	Pear & isodiametric	Dark brown	Ovate
RRPxDSI	Green	Rough	Isodiametric	Brown	Oblong
RRPxNSC	Light green	Smooth	Isodiametric & curvilinear	Brown	Ovate

**Table 3:** Variation in seedling traits at 25 days after planting; vegetative traits and growth traits (50 DAP) among *Lagenaria siceraria* genotypes.

Genotype	DG	EP	SH	CLA	LA	VL	NOL	NOB	LCC	DF	DEM	DHM
KSP	16 ^c^	44.58 ^ef^	3.58 ^b^	12.25	122.9 ^c^	23.70 ^d^	9.17	0.83	20.72	59 ^a^	78 ^d^	119 ^a^
DSI	14 ^d^	66.13 ^a–d^	4.47 ^ab^	12.93	183.4 ^bc^	60.40 ^cd^	12.33	1.33	25.06	57 ^c^	80 ^a^	120 ^a^
NSC	17 ^bc^	45.83 ^def^	4.47 ^ab^	12.81	152.9 ^bc^	75.47 ^bcd^	15.17	2.33	25.62	58 ^ab^	78 ^d^	119 ^a^
NqSC	19 ^a^	32.92 ^f^	5.18 ^ab^	14.63	167.8 ^bc^	111.37 ^abc^	23.83	3.33	24.25	58 ^b^	79 ^b^	119 ^a^
KSR	18 ^b^	49.78 ^c–f^	5.17 ^ab^	15.08	171.6 ^bc^	84.40 ^a–d^	19.17	3.33	23.25	57 ^c^	79 ^c^	119 ^a^
NSRC	9 ^f^	53.47 ^b–f^	4.53 ^ab^	14.14	228.0 ^ab^	108.73 ^abc^	19.17	3.17	22.59	58 ^ab^	79 ^c^	118 ^b^
RRP	9 ^f^	70.62 ^abc^	5.77 ^a^	16.62	310.6 ^a^	143.92 ^a^	24.17	3.67	21.28	56 ^d^	78 ^d^	117 ^c^
NqSCxKSP	12 ^e^	72.33 ^ab^	5.50 ^a^	13.98	175.5 ^bc^	129.35 ^ab^	19.83	3.17	24.64	55 ^e^	78 ^d^	116 ^d^
NqSCxDSI	12 ^e^	67.45 ^abc^	5.40 ^a^	15.33	169.5 ^bc^	86.18 ^a–d^	17.33	2.67	23.84	56 ^e^	79 ^b^	117 ^d^
NqSCxNSC	12 ^e^	67.47 ^abc^	4.53 ^ab^	13.49	169.1 ^bc^	94.43 ^abc^	18.50	3.33	24.21	56 ^e^	78 ^d^	117 ^d^
KSRxKSP	10 ^f^	81.95 ^a^	5.22 ^ab^	15.07	196.3 ^bc^	98.68 ^abc^	17.67	3.17	22.22	55 ^f^	80 ^a^	116 ^e^
KSRxDSI	12 ^e^	*61*.42 ^a–e^	5.20 ^ab^	15.13	179.2 ^bc^	107.07 ^abc^	17.33	3.33	23.57	56 ^e^	79 ^b^	117 ^d^
KSRxNSC	12 ^e^	67.08 ^a–d^	5.32 ^ab^	15.81	177.3 ^bc^	106.17 ^abc^	17.0	2.17	26.73	55 ^e^	75 ^e^	117 ^d^
NSRCxKSP	10 ^f^	70.50 ^abc^	5.30 ^ab^	15.72	206.5 ^bc^	132.90 ^ab^	18.50	3.0	24.49	54 ^f^	74 ^f^	115 ^f^
NSRCxDSI	12 ^e^	68.75 ^abc^	5.63 ^a^	12.93	213.2 ^abc^	117.63 ^abc^	17.67	2.83	26.23	55 ^f^	79 ^b^	117 ^d^
NSRCxNSC	10 ^f^	66.53 ^a–d^	4.47 ^ab^	12.72	151.3 ^bc^	103.05 ^abc^	17.83	2.17	54.51	54 ^f^	78 ^d^	116 ^e^
RRPxKSP	10 ^f^	70.50 ^abc^	5.25 ^ab^	13.63	188.5 ^bc^	119.83 ^abc^	17.67	2.50	25.90	55 ^f^	74 ^f^	116 ^e^
RRPxDSI	13 ^e^	53.50 ^b–f^	5.18 ^ab^	14.45	211.9 ^abc^	110.80 ^abc^	16.83	2.0	25.0	55 ^f^	78 ^d^	117 ^d^
RRPxNSC	12 ^e^	65.97 ^a–e^	4.83 ^ab^	15.65	198.8 ^bc^	109.70 ^abc^	17.0	2.33	22.62	57 ^cd^	78 ^d^	117 ^d^
Significance	***	***	*	ns	***	*	ns	ns	ns	***	***	***
*p*-Value	<0.0001	<0.0001	0.0120	0.4028	0.0002	0.0142	0.7328	0.3606	0.4446	<0.0001	<0.0001	<0.0001
HSD	1.13	21.41	1.80	549.23	11,674	65.20	14.77	3.45	37.0	0.51	0.34	0.49

Variables: DG-Days to emergence, EP-Emergence Percentage (%), SH-Seedling Height (cm), CLA-Cotyledon leaf area (cm^2^), LA-Leaf area (cm^2^), VL-Vine length (cm), NOL-Number of leaves, NOB-Number of branches, LCC-Leaf Chlorophyll content (cci), DF-Days to 50% flowering, DEM-Days to edible harvest maturity and DHM-Days to drying harvest maturity. Means with the same letter within the column are not significantly different (*p* > 0.05). The explanation of genotypes is in Table 1. HSD-Honestly significant difference, CV-Coefficient of variation, MSG-Mean square due to genotype and MSE-Mean square of error. * and *** indicate significant at *p* < 0.05 and *p* < 0.001 respectively, whereas ns indicate non-significance (*p* > 0.05).

**Table 4:** Variation in fruit traits, seed traits, shoot and leaf growth (50 DAP), and yield traits among *Lagenaria siceraria* different genotypes.

Genotype	SF	FM	FR	FP	HSM	TSM	FL	FW	FNL	SL	SW	LG	SG	FY	YP
KSP	144.3	673.9	3.8	1.5	38.8	118.5	16.6	9.3	7.1 ^c–f^	17.7	8.8 ^e^	154.0 ^a^	273.2	6357	0.5
DSI	141.5	1474.6	4.6	1.7	41.0	216.4	15.8	11.9	3.9 ^fg^	18.8	7.5 ^f^	135.7 ^a^	410.4	15,492	1.4
NSC	158.8	863.3	4.2	2.2	46.6	204.5	23.1	11.3	11.6 ^a^	18.2	9.8 ^a–e^	75.6 ^a^	411.6	9685	0.9
NqSC	172.5	1095.8	4.2	2.5	54.4	253.7	21.5	12.7	7.8 ^a–f^	18.0	10.5 ^ab^	43.7 ^a^	329.4	13,462	1.3
KSR	163.0	1331.1	4.8	1.5	55.4	278.3	23.4	12.5	7.3 ^b–f^	18.3	9.2 ^cde^	49.4 ^a^	290.1	13,527	1.3
NSRC	146.3	1917.5	5.0	1.2	57.6	178.3	25.1	13.9	7.1 ^c–f^	20.3	10.3 ^ab^	67.9 ^a^	526.5	9252	0.8
RRP	223.8	1402.0	5.5	1.0	59.2	242.3	18.9	14.3	5.5 ^d–g^	19.0	10.7 ^a^	26.4 ^ab^	177.7	9554	0.9
NqSCxKSP	219.3	1465.0	4.8	1.3	61.9	274.8	26.7	12.2	11.4 ^ab^	19.0	9.4 ^b–e^	23.9 ^b^	242.6	15,123	1.4
NqSCxDSI	164.2	1349.4	4.9	2.0	53.7	303.3	20.4	11.4	6.7 ^c–f^	17.7	9.4 ^b–e^	41.1 ^a^	276.1	15,492	1.4
NqSCxNSC	161.5	958.9	4.5	1.5	53.9	239.1	22.9	10.9	9.3 ^a–d^	19.0	10.0 ^a–d^	88.5 ^a^	402.3	11,016	0.9
KSRxKSP	302.0	2045.3	4.6	1.5	62.2	316.7	27.3	12.9	8.9 ^a–d^	18.3	9.0 ^de^	50.6 ^a^	315.7	13,801	1.3
KSRxDSI	180.7	1414.5	5.3	1.2	56.6	243.1	15.6	13.2	2.1 ^g^	17.5	9.0 ^de^	71.8 ^a^	840.3	9811	0.9
KSRxNSC	152.7	1859.6	4.7	1.3	53.0	206.5	25.5	12.2	8.4 ^a–e^	18.5	9.8 ^ae^	39.6 ^a^	232.5	13,193	1.2
NSRCxKSP	179.5	1152.1	4.0	1.8	50.9	252.7	24.9	12.3	11.5 ^a^	18.8	9.8 ^ae^	51.9 ^a^	470.5	19,472	1.8
NSRCxDSI	203.0	1771.1	5.2	1.2	52.5	191.6	23.7	12.9	7.2 ^b–f^	17.8	9.9 ^a–e^	33.7 ^a^	247.6	23,039	2.1
NSRCxNSC	236.9	1186.1	4.6	1.5	53.8	223.8	23.2	11.7	9.6 ^a–d^	18.3	10.2 ^abc^	126.5 ^a^	292.8	15,682	1.5
RRPxKSP	257.0	1620.6	4.7	1.7	51.9	319.5	25.9	13.1	10.1 ^abc^	18.7	9.5 ^b–e^	27.7 ^ab^	460.0	11,185	1.1
RRPxDSI	214.8	2342.2	5.3	1.2	61.9	236.4	20.9	15.3	4.2 ^e–g^	15.3	9.8 ^a–e^	26.2 ^ab^	249.1	17,022	1.6
RRPxNSC	159.3	1037.3	4.4	1.3	43.2	244.1	18.3	11.8	7.6 ^a–f^	17.0	9.0 ^de^	60.6 ^a^	246.1	6357	0.9
Significance	ns	ns	ns	ns	ns	ns	ns	ns	**	ns	***	*	ns	ns	ns
*p*-Value	0.778	0.087	0.082	0.863	0.099	0.668	0.119	0.256	0.0027	0.135	<0.0001	0.043	0.332	0.456	0.498
HSD	228.3	1468.2	1.7	1.4	28.2	261.4	8.7	3.7	4.2	4.1	1.1	131.4	690.2	17,866	1.7

Variables: SF-Number of seeds per fruit, FM-Fruit mass (g), FR-Fruit rind (mm), FP-Number of fruits per plant, HSM-100 seed mass (g), TSM-Total seed mass (g), FL-Fruit length (cm), FW-Fruit width (cm), FNL-Fruit neck length (cm), SL-Seed length (mm), SW-Seed width (mm), LG-Leaf growth%, SG-Shoot growth%, FY-Fruit yield (kg/ha) and YP-fruit Yield per plant (kg). Means with the same letter within the column are not significantly different (*p* < 0.05). The description of genotypes is in Table 1. HSD-Honestly significant difference. *, ** and *** indicate significant at *p* < 0.05, *p* < 0.01 and *p* < 0.001 respectively, whereas ns indicate non-significance (*p* > 0.05).

**Table 5:** Correlation among morpho-agronomic traits of *Lagenaria siceraria* genotypes.

Variables	EP	SH	CLA	LA	VL	NOL	NOB	LCC	TFM	SF	FR	FP	HSM	TSM	FL	FW	FNL	SL	SW	LG	SG	FY
SH	0.43 ^ns^																					
CLA	0.26 ^ns^	0.66 **																				
LA	0.35 ^ns^	0.58 **	0.57 *																			
VL	0.41 ^ns^	0.79 ***	0.57 *	0.68 **																		
NOL	0.06 ^ns^	0.69 **	0.58 **	0.57 *	0.81 ***																	
NOB	0.18 ^ns^	0.65 **	0.55 *	0.48 *	0.69 ***	0.87 **																
LCC	0.11 ^ns^	−0.17 ^ns^	−0.35 ^ns^	−0.27 ^ns^	0.07 ^ns^	0.01 ^ns^	−0.18 ^ns^															
TFM	0.28 ^ns^	0.14 ^ns^	−0.28 ^ns^	−0.08 ^ns^	0.001 ^ns^	−0.23 ^ns^	−0.21 ^ns^	0.13 ^ns^														
SF	0.54 *	0.43 ^ns^	0.09 ^ns^	0.27 ^ns^	0.45 *	0.31 ^ns^	0.28 ^ns^	0.26 ^ns^	0.01 ^ns^													
FR	0.31 ^ns^	0.59 **	0.34 ^ns^	0.62 **	0.48 *	0.40 ^ns^	0.42 ^ns^	−0.05 ^ns^	−0.14 ^ns^	0.26 ^ns^												
FP	−0.44 *	−0.16 ^ns^	−0.17 ^ns^	−0.50 *	−0.26 ^ns^	0.01 ^ns^	−0.07 ^ns^	0.03 ^ns^	0.09 ^ns^	−0.19 ^ns^	−0.63 **											
HSM	0.26 ^ns^	0.64 **	0.42 ^ns^	0.43 ^ns^	0.65 **	0.68 **	0.70 ***	0.03 ^ns^	−0.08 ^ns^	0.58 **	0.62 **	−0.30 ^ns^										
TSM	0.45 *	0.62 **	0.46 *	0.15 ^ns^	0.46 *	0.47 *	0.51 *	−0.04 ^ns^	−0.11 ^ns^	0.57 *	0.23 ^ns^	0.21 ^ns^	0.49 *									
FL	0.29 ^ns^	0.33 ^ns^	0.04 ^ns^	0.04 ^ns^	0.43 ^ns^	0.36 ^ns^	0.3 ^ns^	0.15 ^ns^	0.29 ^ns^	0.46 *	−0.06 ^ns^	0.06 ^ns^	0.51 *	0.37 ^ns^								
FW	0.11 ^ns^	0.62 **	0.45 *	0.73 ***	0.68 **	0.57 *	0.46 *	−0.11 ^ns^	−0.09 ^ns^	0.40 ^ns^	0.73 ***	−0.40 ^ns^	0.69 **	0.29 ^ns^	0.18 ^ns^							
FNL	0.14 ^ns^	−0.04 ^ns^	−0.18 ^ns^	−0.26 ^ns^	0.16 ^ns^	0.11 ^ns^	0.11 ^ns^	0.22 ^ns^	0.24 ^ns^	0.21 ^ns^	−0.57 *	0.39 ^ns^	−0.01 ^ns^	0.18 ^ns^	0.74 ***	−0.36 ^ns^						
SL	0.22 ^ns^	−0.05 ^ns^	−0.04 ^ns^	0.17 ^ns^	0.13 ^ns^	0.25 ^ns^	0.34 ^ns^	0.01 ^ns^	−0.06 ^ns^	−0.06 ^ns^	−0.09 ^ns^	0.02 ^ns^	0.04 ^ns^	−0.02 ^ns^	0.38 ^ns^	−0.13 ^ns^	0.37 ^ns^					
SW	−0.17 ^ns^	0.32 ^ns^	0.22 ^ns^	0.37 ^ns^	0.58 **	0.70 ***	0.53 *	0.20 ^ns^	−0.11 ^ns^	0.17 ^ns^	0.19 ^ns^	0.003 ^ns^	0.49 *	−0.03 ^ns^	0.43 ^ns^	0.35 ^ns^	0.31 ^ns^	0.15 ^ns^				
LG	−0.22 ^ns^	−0.89 ***	−0.62 **	−0.53 *	−0.77 ***	−0.68 **	−0.63 **	0.32 ^ns^	−0.02 ^ns^	−0.37 ^ns^	−0.49 *	0.10 ^ns^	−0.67 **	−0.58 **	−0.49 *	−0.65 **	−0.12 ^ns^	0.10 ^ns^	−0.45 *			
SG	−0.07 ^ns^	−0.14 ^ns^	−0.06 ^ns^	−0.13 ^ns^	−0.01 ^ns^	−0.09 ^ns^	0.19 ^ns^	−0.08 ^ns^	−0.26 ^ns^	−0.14 ^ns^	0.08 ^ns^	0.04 ^ns^	−0.01 ^ns^	0.02 ^ns^	−0.19 ^ns^	0.09 ^ns^	−0.24 ^ns^	0.17 ^ns^	−0.18 ^ns^	0.18 ^ns^		
FY	0.29 ^ns^	0.49*	−0.06 ^ns^	0.08 ^ns^	0.31 ^ns^	0.15 ^ns^	0.09 ^ns^	0.27 ^ns^	0.84 ***	0.25 ^ns^	0.18 ^ns^	0.06 ^ns^	0.29 ^ns^	0.20 ^ns^	0.35 ^ns^	0.25 ^ns^	0.09 ^ns^	−0.11 ^ns^	0.08 ^ns^	−0.29 ^ns^	−0.18 ^ns^	

Note: *, ** and *** significant Correlation at *p* < 0.05, *p* < 0.01 and *p* < 0.001 respectively and ^ns^—no significant correlation. High correlations have value > 0.6. Variables: EP-Emergence Percentage (%), SH-Seedling Height (cm), CLA-Cotyledon leaf area (cm^2^), LA-Leaf area (cm^2^), VL-Vine length (cm), NOL-Number of leaves, NOB-Number of branches, LCC-Leaf Chlorophyll content (cci), TFM-Total fruit mass per plot (kg), SF-Number of seeds per fruit, FR-Fruit rind (mm), FP-Number of fruits per plant, HSM-100 seed mass (g), TSM-Total seed mass (g), FL-Fruit length (cm), FW-Fruit width (cm), FNL-Fruit neck length (cm), SL-Seed length (mm), SW-Seed width (mm), LG-Leaf growth%, SG-Shoot growth% and FY-Fruit yield (kg/ha).

**Table 6:** Loadings of the variables for the first five principal components.

Variables	PC1	PC2	PC3	PC4	PC5
DG	−0.20	0.07	0.10	0.43	−0.12
EP	0.19	−0.18	−0.15	−0.32	−0.16
SH	0.28	0.05	0.01	0.18	−0.17
CLA	0.18	0.22	0.07	−0.01	−0.29
LA	0.21	0.22	−0.07	−0.06	0.17
VL	0.29	0.04	0.12	−0.02	0.07
NOL	0.23	0.17	0.26	0.13	0.09
NOB	0.22	0.18	0.24	0.05	−0.01
LCC	0.01	−0.26	−0.01	−0.09	0.29
DF	−0.23	0.28	0.13	0.09	0.12
DEM	−0.07	0.16	−0.21	0.20	0.06
DHM	−0.23	0.21	0.04	0.25	0.15
TFM	0.04	−0.36	−0.16	0.25	0.13
SF	0.20	−0.13	−0.03	−0.13	−0.09
FM	0.21	0.02	−0.27	0.06	0.09
FR	0.20	0.22	−0.27	0.003	0.12
FP	−0.11	−0.12	0.31	0.29	−0.24
HSM	0.26	0.09	0.03	0.06	0.09
TSM	0.19	−0.03	0.11	0.02	−0.49
FL	0.17	−0.22	0.27	−0.004	0.14
FW	0.24	0.20	−0.14	0.10	0.13
FNL	0.02	−0.29	0.41	−0.07	0.04
SL	0.02	0.003	0.27	−0.23	0.27
SW	0.15	0.06	0.29	0.07	0.41
LG	−0.26	−0.09	−0.11	−0.19	0.14
SG	−0.03	0.11	−0.02	−0.19	−0.12
FY	0.15	−0.29	−0.15	0.33	0.09
YP	0.16	−0.28	−0.15	0.33	0.05
Eigenvalue	9.65	4.05	3.13	2.45	1.67
Variability%	34.46	14.48	11.19	8.74	5.97
Cumulative%	34.46	48.94	60.13	68.87	74.85

Note: PC1-5: Principal components 1–5. Variables: DG- Days to emergence, EP-Emergence Percentage (%), SH-Seedling Height (cm), CLA-Cotyledon leaf area (mm^2^), LA-Leaf area (cm^2^), VL-Vine length (cm), NOL-Number of leaves, NOB-Number of branches, LCC-Leaf Chlorophyll content (cci), DF-Days to 50% flowering, DEM-Days to edible harvest maturity and DHM-Days to drying harvest maturity, TFM-Total fruit mass per plot (kg), SF-Number of seeds per fruit, FM-Fruit mass (g), FR-Fruit rind (mm), FP-Number of fruits per plant, HSM-100 seed mass (g), TSM-Total seed mass (g), FL-Fruit length (cm), FW-Fruit width (cm), FNL-Fruit neck length (cm), SL-Seed length (mm), SW-Seed width (mm), LG-Leaf growth %, SG-Shoot growth %, FY-Fruit yield (kg/ha) and YP-Yield per plant (kg).

**Table 7:** Genetic parameters for agronomic traits of *Lagenaria siceraria* genotypes.

Variable	δ^2^g	δ^2^e	δ^2^p	PCV	GCV	H^2^	GA
DG	9.04	0.29	54.8	59.9	24.35	16.5	0.84
EP	127.93	103.45	974.5	50.4	18.26	13.1	0.75
SH	0.16	0.73	2.5	31.3	8.0	6.7	0.54
CLA	9082.52	68,080.1	190,655.3	26.8	5.85	4.8	0.45
LA	14,188,463.3	30,758,151	146,647,082	56.6	17.31	9.7	0.64
VL	591.16	959.46	5465.9	73.0	24.01	10.8	0.68
NOL	2.88	49.26	115.8	60.8	9.59	2.5	0.33
NOB	0.11	2.69	6.0	91.9	12.42	1.8	0.28
LCC	0.095	309.02	618.6	97.1	1.20	0.02	0.03
DF	2.27	0.06	13.8	6.6	2.69	16.5	0.84
DEM	2.81	0.03	16.9	5.3	2.15	16.6	0.84
DHM	2.17	0.05	13.1	3.1	1.26	16.5	0.84
TFM	3.57	27.73	76.9	99.2	21.37	4.6	0.44
FM	10,3431.1	486,467.3	159,3521.3	88.9	22.66	6.5	0.53
FR	0.085	0.68	1.9	29.3	6.22	4.8	0.45
FP	0.07	0.46	1.3	75.5	17.29	5.2	0.47
HSM	15.25	179.82	451.1	40.0	7.36	3.4	0.38
FL	10.51	16.97	96.9	44.6	14.68	10.8	0.68
FW	1.22	3.01	13.3	29.4	8.91	9.1	0.62
FNL	6.16	3.95	44.8	86.4	32.02	13.7	0.76
SL	0.39	3.71	9.7	17.1	3.43	3.9	0.41
SW	0.48	0.27	3.5	19.4	7.25	14.1	0.77
LG	833.93	3896.61	12,796.8	179.9	45.93	6.5	0.53
SG	5113.83	107,504.5	245,691.9	140.7	20.29	2.1	0.29
FY	4,279,979	72,038,336	169,756,546	98.4	15.63	2.5	0.33
YP	0.03	0.69	1.6	102.3	14.19	1.9	0.28

Note: δ^2^g-genotypic variance, δ^2^e-environmental variance, δ^2^p-phenotypic variance, GCV-genotypic coefficient of variation, PCV-phenotypic coefficient of variation, H^2^-broad-sense heritability, GA-genetic advancement. Variables: DG-Days to emergence, EP-Emergence Percentage (%), SH-Seedling Height (cm), CLA-Cotyledon leaf area (mm^2^), LA-Leaf area (cm^2^), VL-Vine length (cm), NOL-Number of leaves, NOB-Number of branches, LCC-Leaf Chlorophyll content (cci), DF-Days to 50% flowering, DEM-Days to edible harvest maturity and DHM-Days to drying harvest maturity, TFM-Total fruit mass per plot (kg), FM-Fruit mass (g), FR-Fruit rind (mm), FP-Number of fruits per plant, HSM-100 seed mass (g), FL-Fruit length (cm), FW-Fruit width (cm), FNL-Fruit neck length (cm), SL-Seed length (mm), SW- Seed width (mm), LG-Leaf growth%, SG-Shoot growth%, FY-Fruit yield (kg/ha) and YP-Yield per plant (kg).

**Table 8:** Description of parental landraces according to their origin as well as fruit and seed morphology [9].

PLR	Area	Fruit Color	Fruit Texture	Fruit Shape	Seed Type	Seed Color	Seed Texture	Seed Size	Seed Line	Seed Shape
KSP	Khangelani	Pale green	Smooth	Pear	Asiatica	Brown	Leathery	Large	Present	Slightly oblong to rectangular
KSR	Khangelani	Green	Rough	Isodiametric	Siceraria	Dark brown	Leathery	Large	Present	Slightly oblong to rectangular
DSI	Dundee	Dark green	Smooth	Isodiametric	Siceraria	Dark brown	Smooth	Large	Present	Oblong
RRP	Rorke’s Drift	Pale green	Rough	Pear	Asiatica	Light brown	Leathery	Large	Present	Rectangular
NSC	Nquthu	Pale green	Smooth	Curvilinear	Asiatica	Light brown	Leathery	Medium	Present	Slightly oblong
NSRC	Nquthu	Green	Semi-rough	Curvilinear	Intermediate	Brown	Leathery	Medium	Present	Slightly oblong
NqSC	Nquthu	Pale green	Smooth	Semi-curvilinear	Asiatica	Light brown	Leathery	Medium	Present	Slightly oblong

Note: PLR-Parental Landraces-from Khangelani area (KSP) with smooth pear-shape and (KSR) rough isodiametric shape fruits; from Rorke’s Drift area (RRP) with rough pear-shape; from Nquthu area (NqSC) and (NSC) with smooth curvilinear shape and (NSRC) semi-rough curvilinear shape, and; from Dundee area (DSI) with smooth isodiametric shape.

**Table 9:** Arrangement of North Carolina Design II for crosses.

	Female Parents	KSP	NSC	DSI
Male Parents				
**KSR**		KSRxKSP	KSRxNSC	KSRxDSI
**NSRC**		NSRCxKSP	NSRCxNSC	NSRCxDSI
**NqSC**		NqSCxKSP	NqSCxNSC	NqSCxDSI
**RRP**		RRPxKSP	RRPxNSC	RRPxDSI

Note: Female Parents-smooth pear-shaped KSP from Khangelani area, smooth curvilinear-shaped NSC from Nquthu area, and smooth isodiametric-shaped DSI from Dundee area. Male parents-rough pear-shaped KSR from Khangelani, smooth curvilinear-shaped NSRC and smooth semi-curvilinear-shaped NqSC from Nquthu, and rough isodiametric-shaped RRP from Rorke’s Drift area.

## Data Availability

The data presented in this study are available on request from the corresponding author.
